# The effect of a pelvic compression belt on postural stability in postpartum women

**DOI:** 10.1007/s12283-025-00516-5

**Published:** 2025-07-28

**Authors:** Rachael F. Vatter, Diana Segura-Velandia, Isabel S. Moore, Aimée C. Mears

**Affiliations:** 1 Sports Technology Institute, Wolfson School of Mechanical, Electrical and Manufacturing Engineering, Loughborough University, Loughborough, UK; 2 Wolfson School of Mechanical, Electrical and Manufacturing Engineering, Loughborough University, Loughborough, UK; 3 Cardiff School of Sport and Health Sciences, Cardiff Metropolitan University, Cardiff, UK

**Keywords:** Postpartum, Compression, Stability, Physical activity

## Abstract

The health benefits of physical activity are well known, however, for the postpartum population there are barriers to retuning to physical activity such as pelvic pain and a fear of movement. Pelvic pain can manifest from instability in the pelvic region and lead to impaired balance and postural stability, exacerbating fear of movement. This study aimed to assess the effect of pelvic compression on postural stability in postpartum women and a nulligravida control cohort. The participants’ postural stability was measured using an inertial measurement unit, and the outcome measures JERK, mean velocity, trajectory area index, and root mean squared acceleration calculated, across two visual conditions (eyes open and eyes closed) during tandem and unilateral stances with and without the use of a pelvic compression belt. Significant improvements were observed, particularly in root mean square acceleration (*p* = 0.003) and JERK (*p* = 0.001), when a compression belt was used indicating enhanced postural stability, with the participants moving more smoothly and less intensely when maintaining balance. The effect of compression was highly individualised, suggesting pelvic compression could serve as an effective intervention to improve postural stability, though individual responses warrant a tailored approach for optimal results.

## Introduction

Regular physical activity can provide multiple health benefits for the general population such as reduced risk of chronic diseases [1] and improved mental health [2]. Additionally, engaging in physical activity can help foster social connections and boost self-esteem [3]. These benefits can be particularly pertinent for women during pregnancy and postpartum where physical activity can improve maternal health outcomes whilst also fostering well-being for their new-borns. However, pregnancy and postpartum is a time when women’s engagement in physical activity tends to decrease and given the major musculoskeletal, biomechanical, and psychological changes experienced during pregnancy and childbirth, remaining physically active or reengaging in physical activity post childbirth may be challenging [4].

There have been several barriers to physical activity identified for postpartum women which include lifestyle changes [5, 6], childcare responsibilities [7, 8], as well as physical and psychological [9] barriers such as pain or discomfort [10]. Amongst the physical barriers, pelvic pain is one of the most common issues during postpartum [11–14]. Pelvic pain (defined as a persistent pain in the pelvis or lower abdominals for three months or more [15]), and fear of movement or injury risk are two frequently raised barriers for postpartum women returning to physical activity [4, 7, 16, 17]. Whilst pelvic pain must be viewed from a biopsychosocial perspective considering the possible biological, psychological, and social factors involved [17–21], a contributing factor to pelvic pain, which can be acutely addressed, is proposed to be instability in the sacroiliac (SI) joints and its supporting structures [22–25]. Instability in the SI joint can contribute to impaired balance [26], increasing risk of falls and in turn increasing injury risk. During pregnancy changes such as an increased body mass and an anterior shift in the centre of gravity can affect joint proprioception [27], further impacting balance (defined as the ability to maintain the body’s centre of mass over its base of support, both during static and dynamic conditions). Many of the physiological changes that occur during pregnancy persist into the postpartum period, potentially reducing balance in postpartum women as well [28]. Significant recovery from these physiological changes is still ongoing at 6 weeks postpartum, after which the recovery process slows, and changes become more gradual [29], possibly highlighting that reduced postural stability (defined as ability to maintain a stable upright position whilst stationary [30]) would be more pronounced earlier in the postpartum recovery period. Although the changes during recovery become more gradual after 6 weeks the body is still undergoing recovery and many hormonal, physiological, and anatomical changes persist far into the postpartum period. For example, the displacement of the centre of gravity and postural changes to the spine and hips during pregnancy can cause postural stability issues, which can persist until 6 months postpartum [31]. Issues concerning the pelvic floor such as denervation, usually recover by 12 months postpartum [32], however, in more severe cases denervation can persist far into the postpartum period [33]. Delivery methods and their subsequent recoveries may also contribute to reduced balance, due to the effect of delivery mode on the pelvic floor muscles. A well-functioning pelvic floor contributes to better balance and overall stability [34], with research finding that women who had caesarean deliveries had stronger pelvic floor muscles than those who delivered vaginally [35]. Impaired static balance during the postpartum period can increase the risk of falls, further exacerbating existing barriers to physical activity, such as fear of movement [4]. Given the critical role of balance in physical activity and injury prevention, developing effective strategies to enhance postural stability during this period is essential.

One proposed intervention to improve structural stability of the SI joint is the use of pelvic belts [36]. These belts apply an external force to the pelvic region, enhancing force closure, limiting SI joint movement, and stabilising the pelvic ring [37, 38] (the bony structure that forms the base of the spine, connects the lower extremities to the abdomen, and transfers the weight from the spine to the lower limbs). Pelvic belts sit around the hips and provide external force to the pelvic region, mainly restricting movements of the SI joints [37, 38]. The SI joints usually have very little movement however become lax during pregnancy due to heightened levels of oestrogen and relaxin [39–41]. Doppler imaging has been used to assess the laxity of the SI joints as well as the influence of a pelvic belt on this laxity [30, 31]. Both the effect of introducing a pelvic belt and the force exerted by the belt were investigated. It was found that mean joint laxity decreased with a pelvic belt versus without a belt, with 50 N of force being adequate to decrease the laxity of the SI joints, and forces up to and exceeding 100 N having no further benefits [37, 38]. Studies have demonstrated the effectiveness of pelvic compression belts in improving structural stability of the pelvis [36]. With Cha et al. finding that a pelvic compression belt positioned below the anterior superior iliac spines improved pelvic joint stability and altered control of the lumbopelvic muscles [36]. Postural stability has also been shown to improve with the use of both compression garments and compression belts. Michael et al. found that postural stability was improved when female athletes wore compression garments in comparison to lose fitting shorts [42], and Cakmak et al. found that a maternity support belt significantly improved postural stability in pregnant women [43]. Whilst these studies highlight the effectiveness of pelvic compression in improving both structural and postural stability, all of the mentioned studies omit to report the amount of compression elicited, potentially causing ambiguity in results. Also this approach has not been investigated in a postpartum population. Given the unique physiological changes and increased susceptibility to SI joint instability in the postpartum population, it is hypothesised that external compression to the pelvic region may have a greater positive effect on postpartum women’s postural stability than that of the general populations. This highlights the potential effectiveness of pelvic belts in enhancing postpartum women’s postural stability, which could help them be more comfortable and confident to restart physical activity [44].

The study aims to investigate the effect of pelvic compression belts on static postural stability in postpartum women, as well as a nulligravida (never pregnant) control group. Using an inertial measurement unit (IMU) to quantify stability metrics, the study evaluates the influence of compression on postural stability under different visual and stance conditions. It is hypothesised that postpartum women will exhibit greater improvements in postural stability with the addition of pelvic compression compared to the nulligravida group, thereby highlighting the potential of this intervention to mitigate barriers to physical activity and facilitate postpartum recovery.

## Methods

### Study design, sample size and eligibility criteria

The experimental study used a repeated-measures, cross comparison design to evaluate the effects of pelvic compression on postural stability. This design ensured all participants remained throughout the study and there were no dropouts. Ethical approval was granted by Loughborough University Ethics Board (Ref:14,233).

Prior to participant recruitment, a power calculation was performed to determine appropriate sample sizes for both postpartum and control groups. For postpartum participants, an effect size of 0.6 and standard deviation of 1.0 were derived from prior work [38] yielding a required sample size of 22 participants. For the control group, a sample size of 13 was calculated based on results from similar studies [45]. For postpartum participants to be included in the study they must have: been aged between 18 and 45, given birth in the past 2 years, had a self-reported non-traumatic birth/pregnancy, and experienced no postpartum complications such as postpartum depression, cardiovascular disease, infection, or sepsis. Control participants were required to be nulliparous females aged 18–45. The exclusion criteria for all participants included lower limb injuries or surgeries in the past six months or any balance impairment disorders, such as vertigo, that may influence postural stability.

### Recruitment process and participants

Participants were recruited between February 2024 and November 2024. Postpartum participants were recruited through advertisements shared at local mother and baby groups and the lead investigator’s private social media. Control participants were recruited via local advertisements in Loughborough and the surrounding areas. Twenty-two postpartum women (aged 30–43 years), and 13 control participants (aged 26–45 years) completed the study. All participants provided written informed consent prior to data collection.

### Data collection

To increase participant recruitment and retention, testing took place at local venues hosting mother and baby groups or at the indoor lab at the Sports Technology Institute, Loughborough University. Before commencing the study, postpartum participants completed a questionnaire detailing pregnancy information (e.g. delivery date and mode of delivery), physical activity habits before and after pregnancy, and if they experienced pelvic or lower back discomfort. Mode of delivery information was collected to investigate if there was a correlation between delivery mode and effect of pelvic compression. Control participants completed a demographic questionnaire covering physical activity habits and any reported pelvic or lower back discomfort.

### Protocol

All participants were asked to wear close fitting but non-compression clothing to the data collection. Prior to the stability task commencing, participants removed their shoes before height and body mass measurements were taken. An inertial measurement unit (IMU; Movella DOT, Movella Inc., USA) was positioned on the lower back approximately at the fifth lumbar vertebrae (L5) using double sided adhesive tape. The IMU’s placement was recorded relative to the floor using a stadiometer, with this information the IMU’s local coordinate system was reoriented to align with a global coordinate system using a previously validated mathematical procedure to account for the natural lordosis of the spine [46]. Due to some of the drawbacks of conventional equipment used in balance studies such as their expense [47], and restrictive set-up [48, 49], wearable sensors such as inertial measurement units (IMUs) have started to be used. IMUs are lightweight, low cost and can be used in many environments (avoiding environments with any magnetic interference) [50], and as such their convenience and portability make them well-suited for field-based measurements. The use of wearable sensors such as IMUs for evaluating balance has been rapidly increasing, as evidenced by the growing body of research highlighted in the systematic review completed by Ghislieri [51].

Three different stances, bilateral, tandem, and unilateral, all performed on solid flat ground, adapted from the Balance Error Scoring System [52] were tested under two visual conditions (eyes open, EO; eyes closed, EC) and with and without the use of a pelvic compression belt. Stances were explained to participants via standardised verbal instructions as well as demonstrated, and participants were given a familiarisation period to ensure they understood the directions. For the bilateral stance, the participants were told to stand evenly on both feet, with feet approximately hip width apart. For tandem stance, one foot was placed in front of the other with the toe of the back foot touching the heel of the front foot, with the non-dominant leg in front. Finally, for unilateral stance, the women stood on their non-dominant leg, with the hip of the non-standing leg in a neutral position, and the knee flexed to 90°. The dominant leg for all participants was defined as the leg they would prefer to kick a ball with. During the EO condition, participants focussed on a black cross positioned at eye level, 3 m in front of them. Rest breaks were provided between stances to reduce fatigue, and participants were allowed to steady themselves or stop if they felt unbalanced.

**Fig. 1:**
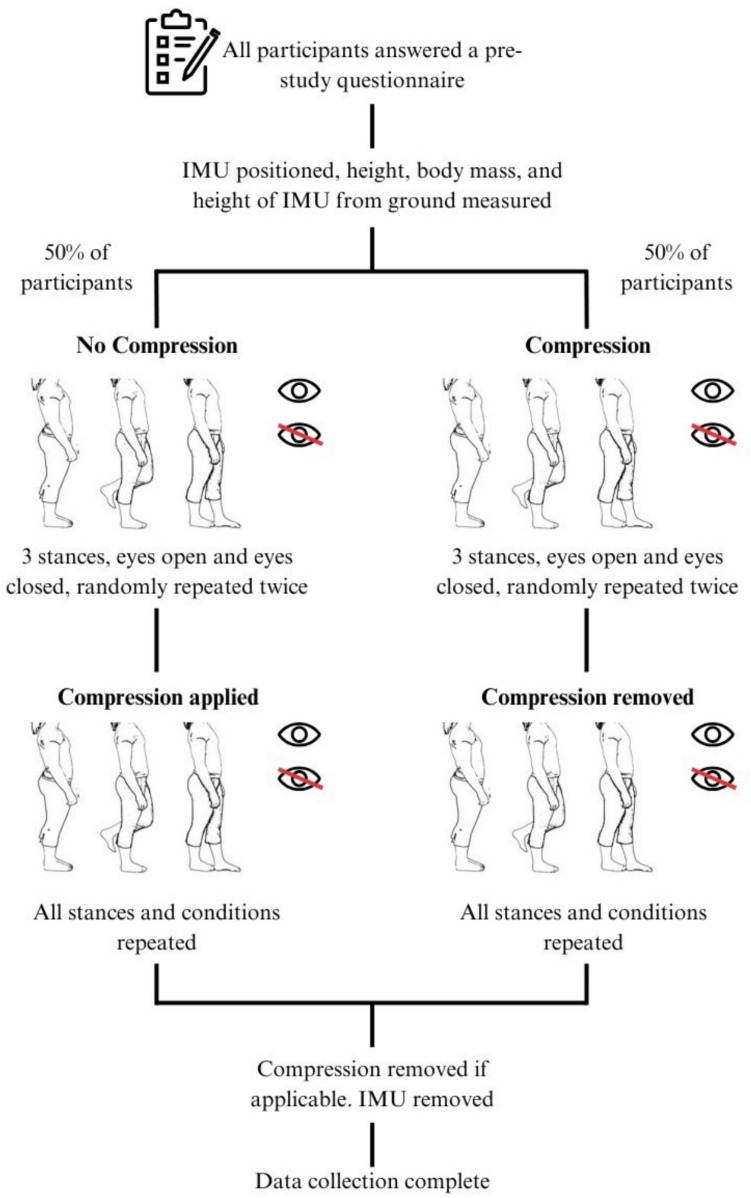
Protocol layout

Half of the participants were tested first in the no-compression condition, followed by the addition of compression and a second round of testing (Fig. 1). For the other 50%, testing began with the compression condition, after which the compression belt was removed, and the stances performed again. The order in which the 12 stances were completed was randomised for both the compression belt and no compression belt conditions. Compression was applied to the pelvic region via a maternity support belt (PhysioRoom®), which could be adjusted between 80 and 120 cm using Velcro fastenings. The physio room band was used as the Velcro fastenings meant the compression applied could be easily adjusted to the desired level. The physio room maternity support belt could also accommodate a range of sizes of participants making it a more inclusive method of supplying compression.

**Fig. 2:**
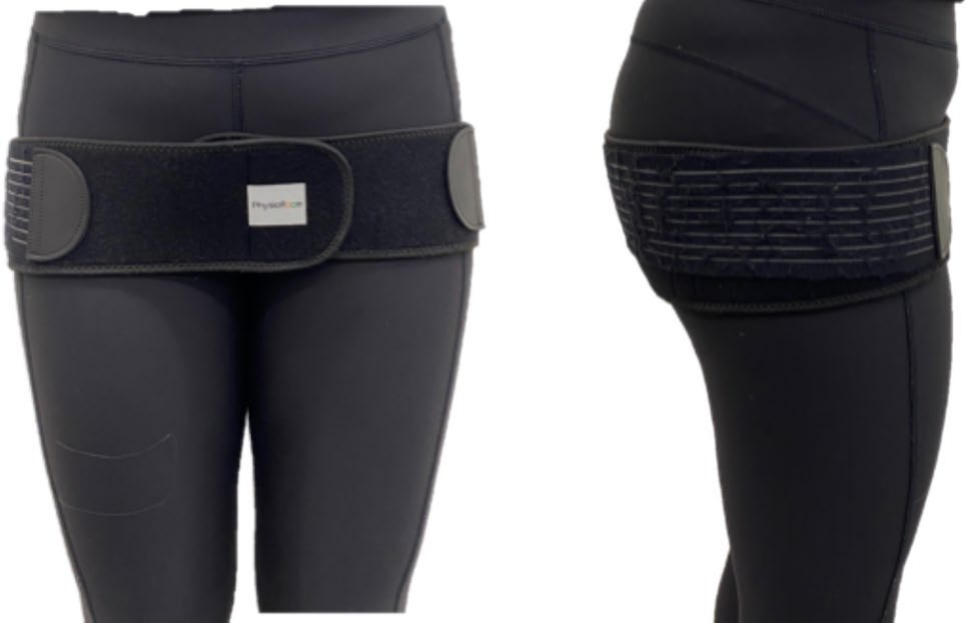
Low position of belt shown on participant

In total, each participant carried out 12 unique stances, each lasting 30 s and repeated twice. Compression was applied via a maternity support belt (PhysioRoom®) worn in the low position (Fig. 2) and adjusted to a level of 30 mmHg for all participants, measured using a Kikuhime pressure monitor. The maternity support belt sat at the level of the pubic symphysis in line with Mens et al. [38].

The Kikuhime sensor was positioned over the greater trochanter, placed on top of the participant’s clothing and beneath the pelvic belt, to ensure consistent compression for all participants.

One IMU was used throughout data collection. The sensor comprised of a tri-axial accelerometer, gyroscope, and magnetometer with respective ranges ± 16 g, ± 2000°/s, and ± 8 Gauss, and a mass of 11.2 g. Acceleration data along three perpendicular axes were recorded at a sampling frequency of 120 Hz, and then exported as a.csv for further analyses.

### Outcome measures

Postural stability was quantified using resultant root mean squared (RMS) acceleration (m/s^2^), which indicated the magnitude of the acceleration [53]. The resultant JERK (m^2^/s^5^) an indicator of the smoothness of the movement, was calculated as the time integral of the square of the magnitude of jerk [54]. Sway velocity (m/s), was calculated by integrating mediolateral (ML) and anteroposterior (AP) accelerations [53], and then finding the resultant value. Trajectory area index (TAI), calculated by finding the area encompassed by the acceleration trajectory plot in the ML and AP directions [53], using equations adapted from [55, 56], was used as an index of overall postural control [51].

The outcome measures were selected to capture different aspects of postural stability. The RMS acceleration reflects how intensely movement is in response to postural corrections and indicates how much the body is moving [57]. JERK assesses smoothness of the movement with increased JERK values showing abrupt and less controlled movements, indicating poor stability [54]. Sway velocity reflects how quickly the body moves during postural adjustments, high sway values indicate less control and more rapid adjustments to maintain stability [58]. The TAI offers a visual representation of the extent of movement during a postural task, with a large TAI indicating poor control of movement [51].

All measures were investigated in the ML, AP directions with resultant values then calculated, all metrics presented are resultant values. All metrics were computed using a custom MATLAB script. Lower values across these measures indicated improved postural stability [53, 55, 59]. Bilateral stance data were excluded from final analyses due to minimal variances between conditions.

### Statistical analysis

Data were analysed using SPSS (Version 29) with statistical significance at *p* < 0.05. A Shapiro–Wilk test was used to determine normality for all outcome measures. Paired t-test (parametric data) and Wilcoxon signed-rank (non-parametric data) were used for within group comparisons of stability metrics between compression conditions. Independent t-tests (parametric data) and Mann–Whitney U tests (non-parametric data) were employed for between group comparisons for control and postpartum groups to investigate relationships of demographic characteristics and influence of compression. Independent t-tests and Mann–Whitney U tests were also employed for between group analysis for delivery mode (vaginal or caesarean) groups. Mixed ANOVA was avoided due to violations of normality and sphericity assumptions.

## Results

### Demographics

Table 1 provides details of demographic characteristics for both postpartum and control cohorts. There was no statistical difference in age, height, or weight between the postpartum and control groups. Fourteen postpartum participants had one child, with the remaining eight having two children. Twelve women had vaginal deliveries, and the remaining ten participants had caesarean section (caesarean) deliveries (Table 1). Four women indicated they experienced pelvic pain, with a further three experiencing pelvic pain during activity. Twenty participants were active prior to pregnancy, 21 were active during pregnancy, and 21 had returned to physical activity since giving birth. Postpartum participants’ activity levels ranged from twice to nine times a week. Participants took part in a range of different activities, with walking, running, weightlifting, high-intensity interval training (HIIT) classes and swimming being the most common. Three participants ceased physical activity between eight to 15 weeks pregnant, with all other participants (*n* = 19) remaining active past 34 weeks. Participants that had returned to physical activity (*n* = 21) returned between 1 and 12 weeks postpartum. Participants returned to day-to-day activities such as walking or taking part in ‘mum fitness’ classes sooner than returning to higher impact/exertion activities such as running or weight training. The control participants were active between once and ten times a week, with the most common physical activities being running, cycling, and swimming.

**Table 1:** Demographic characteristics of postpartum and control participants

	Postpartum participants									Control participants		
	Vaginal delivery (n = 12)			Caesarean delivery (n = 10)			Overall (n = 22)			Overall (n = 13)		
Demographics	Mean	SD	Range	Mean	SD	Range	Mean	SD	Range	Mean	SD	Range
Height (cm)	170.3	7.7	161–191	164.6	5.2	154–172	167.6	7.2	154–191	168.4	6.7	155.5–176.5
Body mass (kg)	75.6	17.4	55.8–108.6	71.3	11.5	50.4–90.1	73.7	15.2	50.4–108.6	63.1	14.2	41–98.8
Age (years)	34.0	3.1	30–41	37.0	3.7	30–43	35.0	3	30–43	32.5	5.3	26–45
Time since birth (months)	10	5	1–18	13	5	5–21	11	5	1–21	–	–	–
Pelvic pain (day-day)	2	–	–	2	–	–	4	–	–	4	–	–
Pelvic pain (during activity)	2	–	–	1	–	–	3	–	–	1	–	–

### Influence of pelvic compression belt on stability metrics

For postpartum participants, significant improvements in postural stability metrics (reduced outcome measures) were observed across all tested conditions (EO and EC) when the pelvic belt was applied (Table 2). Specifically, reductions were noted in RMS acceleration (*p* = 0.010), JERK (*p* = 0.026), mean velocity (*p* = 0.003), and TAI (*p* = 0.013) during tandem stance with EO. Similar trends were observed in unilateral stance, particularly under EC conditions, where reductions in RMS acceleration (*p* = 0.002) and TAI (*p* = 0.004) were most pronounced. The only significant differences found in control participants were during EC conditions, with JERK (t = 2.586, *p* = 0.024) and mean velocity (t = 3.400, p = 0.005) improving during tandem stance, and RMS (t = 3.198, *p* = 0.008) improving during unilateral stance. All other conditions and outcome metrics showed no significance with the addition of the pelvic belt for control participants. Control participants exhibited fewer significant differences between compression and no compression conditions, suggesting postpartum women may derive greater benefits from compression in terms of postural stability.

**Table 2:** Mean values of outcome measures before and after compression (before comp and after comp respectively), and Wilcoxon signed-rank (Z, p) and paired t-test (t, p) results for all outcome measures for tandem and unilateral stances, EO and EC for postpartum participants

	Tandem Stance								Unilateral Stance							
	EO				EC				EO				EC			
	Mean		Z/t	*p*	Mean		Z/t	*p*	Mean		Z/t	*p*	Mean		Z/t	*p*
	Before comp	After comp			Before comp	After comp			Before comp	After comp			Before comp	After comp		
JERK	0.0325	0.0228	−2.224^a^	0.026^*^	0.1320	0.0766	−3.198^a^	0.001^*^	0.0516	0.0441	−2.354^a^	0.019^*^	0.2503	0.2014	3.081	0.006^*^
Mean velocity	0.4510	0.3929	−2.938^a^	0.003^*^	0.7535	0.7053	−2.192^a^	0.028^*^	0.5132	0.4532	−3.133^a^	0.002^*^	0.7891	0.6880	−2.912^a^	0.004^*^
TAI	0.0064	0.0031	−2.484^a^	0.013^*^	0.0234	0.0167	1.632	0.118	0.0066	0.0074	−.536^a^	0.592	0.1302	0.0493	−2.875^a^	0.004^*^
RMS	0.0536	0.0467	−2.581^a^	0.010^*^	0.0824	0.0725	3.312	0.003^*^	0.0618	0.0578	−1.932^a^	0.053	0.1030	0.0869	−3.062^a^	0.002^*^

^a^Represents that Wilcoxon signed-rank test was performed

^*^Statistically significant relationship between influence of compression and outcome measure

**Fig. 3:**
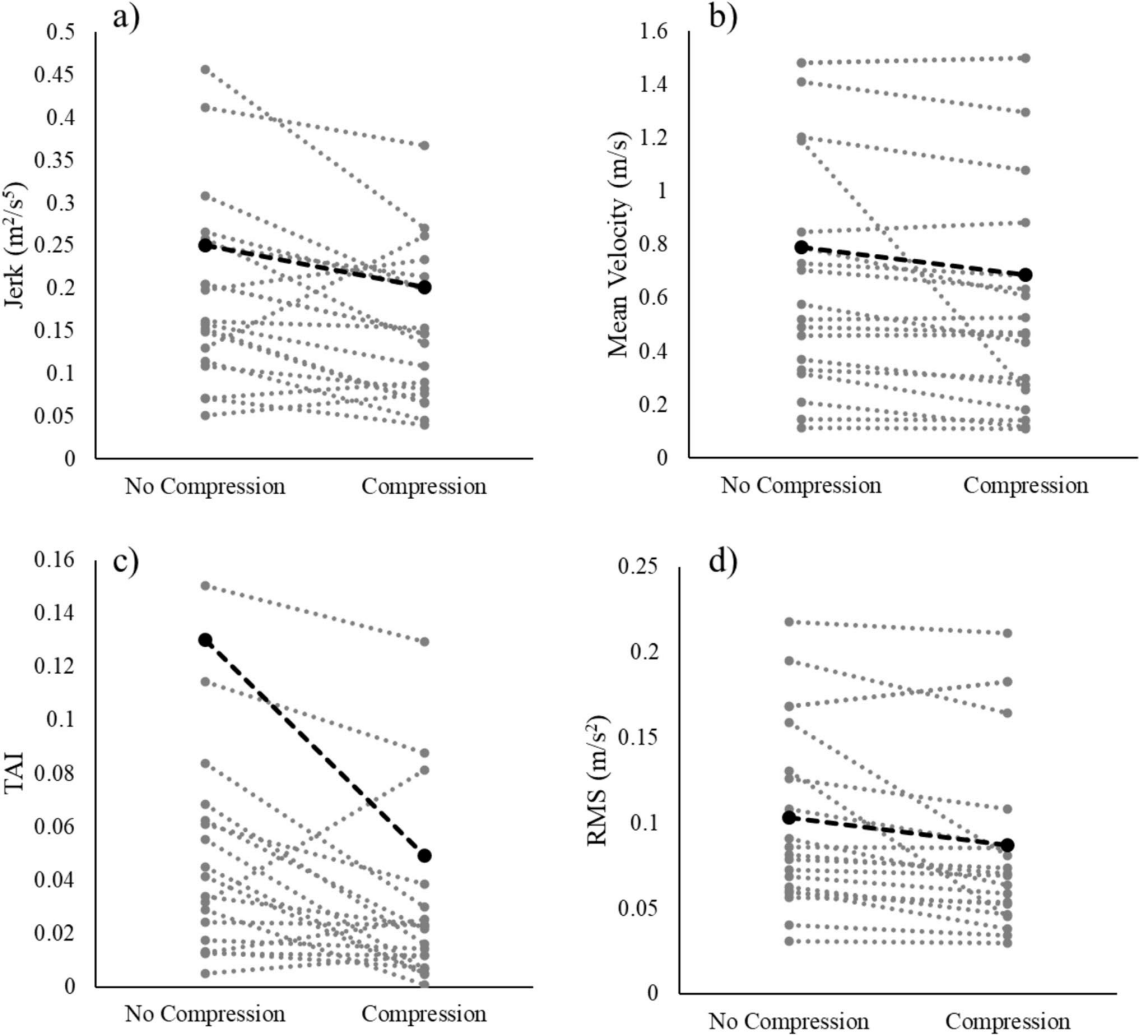
Graphs showing postpartum mean (*n* = 22) of different outcome measures with and without compression (black, dashed), with grey dotted lines showing 20 postpartum participants individual response, during unilateral stance EC

Whilst significant differences were not found for all outcome variables for visual and compression conditions, there was a reduction in the mean value of all outcome variables between no compression and compression conditions for both postpartum and control participants. The effect of compression on outcome measures was very individualised for each participant (Fig. 3).

Postural stability was consistently better in EO compared to EC across both participant groups, as expected. However, the relative improvement with compression was more pronounced under EC conditions, particularly for postpartum participants which suggests that pelvic compression may reduce reliance on visual input for maintaining stability.

**Fig. 4:**
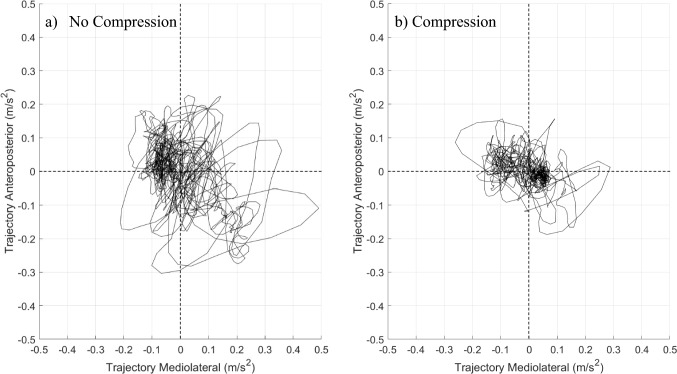
Example from one participant (19 months postpartum) of acceleration trajectory plots showing the acceleration trajectories in mediolateral and anteroposterior directions during unilateral stance, EC, **a** no compression, **b** compression. Dashed line shows 0,0

Acceleration trajectory plots further highlighted the effect of compression, with smaller and less erratic trajectories observed when compression was applied (Fig. 4). Notably participants in early postpartum recovery (< 6 months) demonstrated larger trajectory areas compared to those further into recovery (> 12 months), underscoring the potential timing-dependent benefits of pelvic compression.

**Fig. 5:**
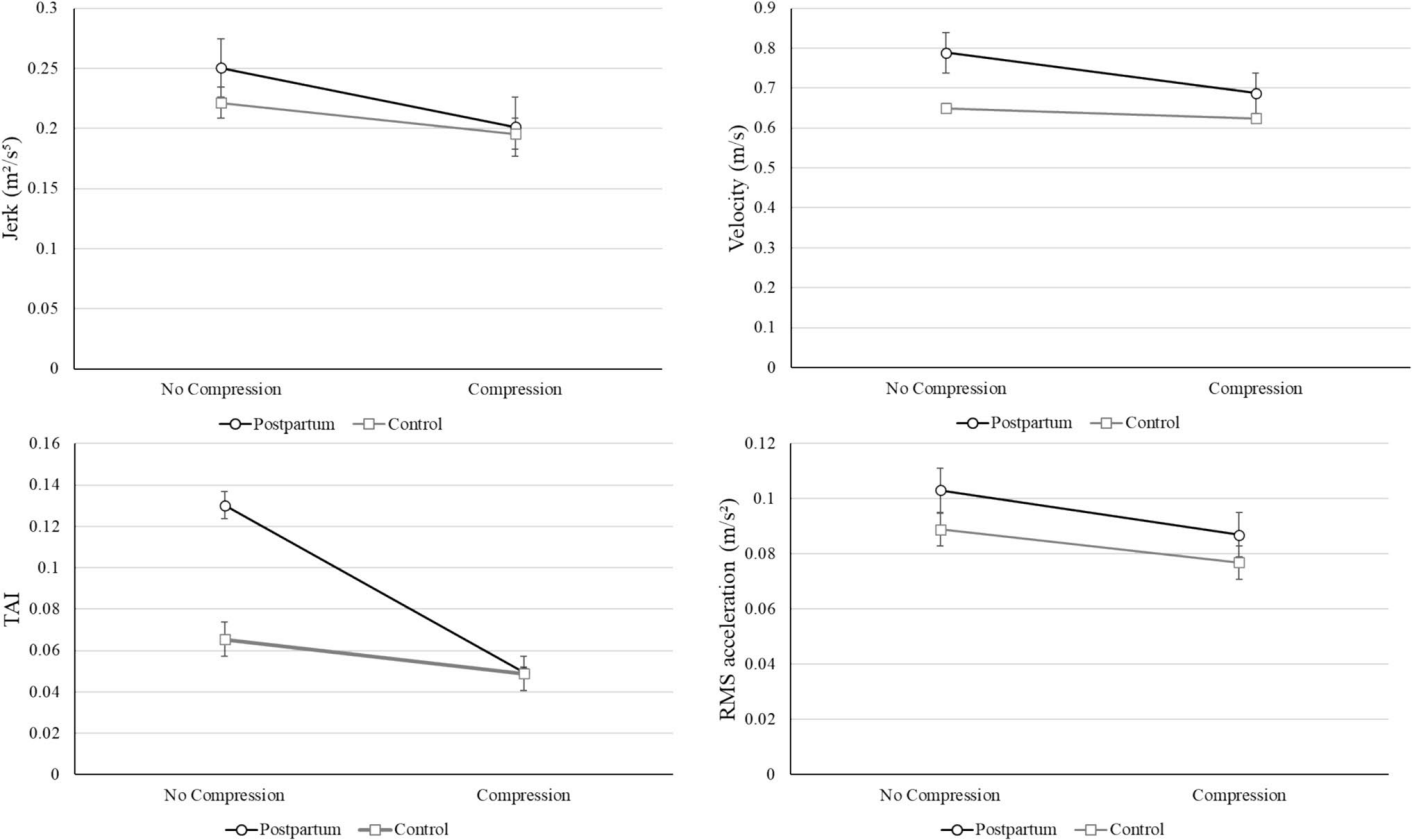
Graphs showing the mean of postpartum (*n* = 22, solid black line) and control (*n* = 13, solid grey line) participants with and without compression during unilateral stance EC, with error bars showing standard error

The mean outcome measure values for postpartum and control participants both decreased with the addition of compression, indicating an increase in postural stability for both cohorts (Fig. 5). However, postpartum participants improved more than control participants, shown by the slope of the graphs in Fig. 4, indicating postpartum women may derive greater benefits from compression in terms of postural stability.

No relationship was found for the between improvement in outcome measures JERK, mean velocity or TAI in either tandem or unilateral stances when compression was applied and delivery mode. Significance was shown, however, between the influence of compression and delivery method for the outcome measure RMS acceleration (Z = -2.078, *p* = 0.038) during tandem stance EO. Indicating that women who experienced a vaginal delivery showed better improvement in postural stability when wearing compression than women who delivered via caesarean.

## Discussion

The aim of this study was to assess the effect of using a pelvic compression belt, on postural stability in postpartum women as well as a nulligravida (never pregnant) control group. The results indicate pelvic compression decreases outcome measures indicating improved postural stability in postpartum women. The significant reductions in JERK, RMS acceleration, and TAI metrics indicate smoother and more controlled movements with compression, aligning with prior findings in general populations [42, 43, 45]. In the present study, pelvic compression led to an improvement in stability outcome variables, this positive effect of pelvic compression is in line with Bey et al. [60], who found a small but significant effect on postural stability in both pregnant and non-pregnant women, their study, however, did not investigate postpartum women. Further studies have also found this positive improvement in stability related to lower body compression garments, both in pregnant women and female athletes [42, 43]. To the authors’ knowledge, this is the first paper to investigate the effect of pelvic compression on postpartum women’s postural stability.

Postpartum participants demonstrated improved postural stability in the EO condition than the EC, as evidenced by reduced JERK, TAI and RMS in both tandem and unilateral stances. These results were expected as vision is a major input to the control of orientation and balance, therefore, by removing the visual cues whilst balancing leads to a more perturbed stance [61, 62]. The lack of statistical significance in mean velocity during tandem stance suggests velocity may not be as sensitive to visual conditions, in accordance with previous research [53].

The findings in the current study, showing improvements in postural stability in both cohorts due to compression, align with previous research indicating that compression affects stability in female athletes under EC conditions [42]. However, unlike the study by Michael et al. [42], the present research found improvements in stability under both EO and EC conditions with compression. Additionally, the compression methods differed between studies; the current study used a pelvic belt, whilst the previous research utilised long leg compression leggings and the difference in the location of compression may explain the contrasting results. Using a pelvic belt allowed more localised compression to be applied, stabilising the SI joints and its supporting structures as instability in these areas are known contributors to impaired balance [26].

The postpartum participants had worse baseline balance than control participants in some stance conditions, however, this was not seen for all stance and visual conditions, similar to findings by Dumke et al. [63]. The individualised responses observed suggest the effectiveness of pelvic compression may depend on factors such as recovery stage, and baseline postural stability. These findings underscore the need for tailored interventions which consider individual differences in biomechanics and postpartum women’s recovery timelines.

There was significance found between the influence of compression and delivery method. Indicating that women who experienced a vaginal delivery showed better improvement in postural stability when wearing compression than women who delivered via caesarean. Improvement was found in the outcome measure RMS acceleration which indicates that the pelvic belt minimised the intensity of postural readjustment. Whilst significance was found, it was only apparent in one of the four outcome measures, which therefore does not represent overall postural stability. The pelvic floor plays a crucial role in stabilising the spine and pelvis, with a well-functioning pelvic floor contributing to better balance and overall stability [34]. Zhao et al. [35] found that the pelvic floor muscles of women who had caesarean deliveries were stronger than women who had had a vaginal delivery. If this also applies to the women in the current study, it could help explain why those who had vaginal deliveries showed greater improvements in RMS acceleration compared to those who underwent caesarean deliveries when wearing a pelvic belt. This suggests that women who underwent vaginal deliveries may have experienced greater pelvic floor weakness leading to a reduced pelvic stabilisation and, consequently, a more substantial benefit from the external support offered by the pelvic belt. Although vaginal delivery is associated with a greater risk of pelvic floor muscle damage compared to a caesarean section, the condition of these muscles can vary based on how they are managed before and after childbirth. Since this study did not assess pelvic floor muscle function, firm conclusions cannot be drawn about balance in relation to the mode of delivery.

The present study investigated supplying external force to the pelvis via a pelvic compression belt, however, other papers have found similar improvements in balance through the use of compression garments [42]. Compression offers support to active muscles, in the present study the glutes and abdominals, as such enhancing proprioception and reducing the body’s reliance on visual input to maintain balance [64, 65]. It is proposed that surface compression stimulates cutaneous mechanoreceptors, providing additional sensory feedback helping improve body awareness in turn contributing to greater joint stability [64, 66]. Amongst control participants, associations between compression and improvements in postural stability were observed only under EC conditions. This suggests that in the absence of visual input, participants relied more heavily on tactile feedback provided by the pelvic belt. Conversely, when visual input was available, EO, the contribution of tactile feedback to postural stability appeared to be diminished. Overall, the present study adds merit to this proposal by showing a pelvic belt can be effective in enhancing postural stability, with reduction in stability metrics most prominent in EC conditions.

Whilst several studies have reported that pelvic compression belts enhance postural stability, other research has shown that whole/lower-body splinting can increase postural sway [67]. This apparent contradiction may be explained by the differences in the mechanisms involved. Pelvic compression belts provide targeted stabilisation to the pelvic region, enhancing proprioceptive feedback and control. Whereas whole/lower-body splinting may restrict natural segmental movement necessary for maintaining balance. Wearing a whole-body splint restricts antiphase trunk motion [68], which is detrimental to balance, as anti-phase coordination between the trunk and the legs has been shown to reduce postural sway [69].

Whilst the current study has shown that pelvic compression applied low on the pelvis via a pelvic belt has a positive impact on postpartum women’s postural stability, the practicality of this type of compression must be considered. The pelvic belt used in this study was bulky and could limit movement around the thighs, possibly hindering daily movement, and causing women to not wear the pelvic belt. This is in line with findings by Flack et al. who found that pregnant women were more likely to wear a flexible rather than rigid pelvic belt [70]. Ho et al. identified aesthetics as one of five major themes which pregnant women prioritise when considering garments [71]. Incorporating the specific magnitude and placement of the compression identified in the present study into functional active wear, such as leggings with integrated pelvic support, may offer a practical and accessible intervention for postpartum women. Embedding compression within garments already worn by this population also addresses aesthetic considerations, thereby enhancing both usability and adherence.

Overall, these findings contribute to the growing evidence supporting pelvic compression as a practical and effective intervention for improving postural stability. This study highlights how these improvements pertain to postpartum women and may help them in their journey in returning to physical activity. By addressing biomechanical barriers to physical activity, this approach has the potential to reduce fall risk by increasing postural stability [72, 73], in turn increase confidence in movement [44], and support the postpartum population in their physical activity journeys.

## Limitations

Given the specific cohort being investigated and the potential barriers they face in participating in research studies, it was important that the study did not take too much time from the postpartum women’s regular routines. As a result, only two repetitions of each stance were performed, and average values recorded. If time had not been a constraint, additional repetitions could have been performed, allowing for more robust means to be calculated. Another limitation of the current study is the potential for participant fatigue, resulting in a decrease in stability over the course of the study, randomisation in the testing order was implemented to limit this. Interesting results such as the effect of compression on women earlier in their postpartum journey were apparent, however, could not be fully realised within the scope of this study. Future research should explore the influence of pelvic compression on postural stability in women early on in their postpartum journey (< 6 months) as well as later in their postpartum journey (> 12 months) to investigate effect of recovery time, on influence of pelvic compression. The participants only completed one visit to the lab, therefore, reproducibility across different time points was not assessed. Whilst significance was found for the influence of pelvic compression on postural stability dependent on delivery method, the study was underpowered for this analysis. Future work should investigate how delivery method may affect postural stability in postpartum women, using a larger cohort than the current study. Additionally, the lack of direct assessment of pelvic floor muscle function limits the ability to fully interpret the relationship between delivery method and postural stability.

## Conclusion

The findings of this study highlight the significant positive impact of pelvic compression on postural stability in postpartum women. The results demonstrate the use of a pelvic compression belt leads to measurable improvements in key stability metrics such as resultant JERK, mean velocity, TAI, RMS acceleration. These improvements were more pronounced in postpartum participants compared to the nulligravida control group, underscoring the utility of pelvic compression as an effective intervention for enhancing postural stability in this population. This study also highlights the individualised nature of responses to compression, emphasising the need for a tailored approach when implementing such interventions. These findings have important implications for postpartum rehabilitation and the design of assistive devices aimed at supporting physical recovery. By enhancing postural stability, pelvic compression belts could play a crucial role in reducing fall risk and facilitating the return to physical activity for postpartum women. 

## Data Availability

No datasets were generated or analysed during the current study.
